# Twelve Weeks of Daily Lentil Consumption Improves Fasting Cholesterol and Postprandial Glucose and Inflammatory Responses—A Randomized Clinical Trial

**DOI:** 10.3390/nu16030419

**Published:** 2024-01-31

**Authors:** Morgan L. Chamberlin, Stephanie M.G. Wilson, Marcy E. Gaston, Wan-Yuan Kuo, Mary P. Miles

**Affiliations:** 1 Department of Food Systems, Nutrition, and Kinesiology, Montana State University, Bozeman, MT 59717, USA; morgan.chamberlin@student.montana.edu (M.L.C.); wanyuan.kuo@montana.edu (W.-Y.K.); 2 United States Department of Agriculture, Agricultural Research Service Western Human Nutrition Research Center, Davis, CA 95616, USA; smgwilson@ucdavis.edu; 3 Texas A&M, Institute for Advancing Health Through Agriculture, College Station, TX 77845, USA; 4 Department of Human Ecology, SUNY Oneonta, Oneonta, NY 13820, USA; marcy.gaston@oneonta.edu

**Keywords:** dietary pulses, dietary fiber, postprandial period, metabolic disease

## Abstract

Lentils have potential to improve metabolic health but there are limited randomized clinical trials evaluating their comprehensive impact on metabolism. The aim of this study was to assess the impact of lentil-based vs. meat-based meals on fasting and postprandial measures of glucose and lipid metabolism and inflammation. Thirty-eight adults with an increased waist circumference (male ≥ 40 inches and female ≥ 35 inches) participated in a 12-week dietary intervention that included seven prepared midday meals totaling either 980 g (LEN) or 0 g (CON) of cooked green lentils per week. Linear models were used to assess changes in fasting and postprandial markers from pre- to post-intervention by meal group. Gastrointestinal (GI) symptoms were assessed through a survey randomly delivered once per week during the intervention. We found that regular consumption of lentils lowered fasting LDL (F = 5.53, *p* = 0.02) and total cholesterol levels (F = 8.64, *p* < 0.01) as well as postprandial glucose (β = −0.99, *p* = 0.01), IL-17 (β = −0.68, *p* = 0.04), and IL-1β (β = −0.70, *p* = 0.03) responses. GI symptoms were not different by meal group and all symptoms were reported as “none” or “mild” for the duration of the intervention. Our results suggest that daily lentil consumption may be helpful in lowering cholesterol and postprandial glycemic and inflammatory responses without causing GI stress. This information further informs the development of pulse-based dietary strategies to lower disease risk and to slow or reverse metabolic disease progression in at-risk populations.

## 1. Introduction

Obesity-related diseases such as nonalcoholic fatty liver disease, type 2 diabetes, and cardiovascular disease are among the leading causes of morbidity and mortality worldwide [1]. These diseases are characterized by abnormal glucose and lipid metabolism driven by biological and environmental factors including genetic predisposition, lifestyle, and diet [1]. Increased central adiposity, in particular, drives metabolic dysregulation through the promotion of macrophage infiltration, low-grade inflammation, altered lipid metabolism, and insulin resistance [2,3,4]. Diet is a known contributor to central adiposity and metabolic dysregulation. Therefore, healthy dietary changes represent one strategy to prevent and reduce the burden of metabolic disease. 

Pulse crops have potential to improve metabolic health [5,6,7,8,9,10] as they are a type of legume that offers consumers a low-cost, plant-based protein, rich in fiber with low fat and sodium content. Pulse protein content ranges between 17% and 30% (dry weight) [11] and provides up to 30 g of fiber per 100 g dry weight, around half of which is insoluble fiber [12]. Fiber offers various metabolic health benefits including increased satiety [13], reduced triglyceride absorption, and improved glycemic control [14,15]. Increased fiber intake has also been linked to blunted systemic inflammation [16,17], which often manifests with altered lipid and glucose metabolism. However, metabolic benefits vary by pulse variety, dosage, and duration of inclusion in the diet [15]. As such, comprehensive dietary intervention studies examining inflammatory and metabolic responses to individual pulse consumption are needed to devise effective dietary recommendations regarding pulse consumption for metabolic health improvements. In addition, gastrointestinal (GI) symptoms are commonly reported as a barrier to pulse consumption [18], yet are rarely reported in pulse intervention studies. Increased transparency of reported GI symptoms may help encourage and increase pulse consumption among consumers.

Lentils are a type of pulse known for their unique nutritional profile. They are high in dietary fiber and protein and contain dietary bioactive compounds such as polyphenols [19]. Randomized clinical trials found improvements to measures of glycemic control including postprandial glucose, glycated hemoglobin (HbA1c), and the homeostatic model of insulin resistance (HOMA-IR) with lentil consumption [8,9,19,20]. However, there is a lack of studies assessing more comprehensive measures of metabolic health including postprandial lipid metabolism and systemic inflammation. Postprandial lipid [21,22,23] and glycemic responses [24] are better predictors of disease risk than fasting levels and are also causally linked to low-grade inflammation [25,26], which drives metabolic disease progression. Although these measures are more robust in predictive power, their assessment is not yet established in the clinical setting. They are, however, excellent markers to better describe metabolic health in the research setting. Individuals with these conditions therefore represent an ideal target population to examine how dietary interventions may be used to prevent disease progression.

We strategically conducted this clinical trial with individuals at high-risk for metabolic disease as defined by an elevated waist circumference, as an accepted proxy for central adiposity [4], and increased postprandial triglyceride response. The aim of this study was to determine the impact of 12 weeks of lentil consumption on fasting and postprandial measures of lipid metabolism, glycemic control, and inflammation. Few studies have investigated the long-term impact of lentil consumption at the recommended dose set by the US Department of Agriculture (USDA), which suggests approximately 1.5 cups (~300 g) of cooked pulses per week for adults with a daily caloric intake of 2000 calories (kcal) [7,27]. For this reason, we investigated a daily lentil dose exceeding this recommendation at 980 g per week which aligns with study doses from publications reporting improvements in blood lipid profiles, inflammation biomarkers, and other metabolic markers from daily pulse consumption [7]. In addition, we conducted weekly surveys during the intervention to assess how lentil consumption at this dose impacts GI symptoms and satiety. This is the first study that we know of to assess dynamic lipidemic, glycemic, and inflammation responses in this population to demonstrate new and potentially more meaningful health impacts of long-term lentil consumption. 

## 2. Materials and Methods

### 2.1. Ethics Statement

The study protocol was approved by the Institutional Review Board at Montana State University (2020-131-MM010320-FCR). The informed consent was reviewed verbally in-person, allowing time for questions and clarifications. Written informed consent was obtained from all participants prior to their participation. This study was prospectively registered with ClinicalTrials.gov (NCT04283448). 

### 2.2. Study Population

Potential participants were recruited via advertisement from June 2020 to the end of August 2023 and screened for eligibility using REDCap (version 13.10.6) [28,29] electronic data capture tools hosted at Montana State University, Bozeman. Inclusion criteria included being between 18 and 70 years old, having a waist circumference ≥ 40 inches for males or ≥35 inches for females, and having a non-fasting serum triglyceride concentration greater than 1.69 mmol/L (150 mg/dL). These criteria were implemented in an effort to recruit individuals with a greater risk of chronic disease development [4]. Potential participants were excluded if they had taken oral antibiotics within 90 days of study enrollment, regularly used anti-inflammatory, oral contraceptive, or lipid lowering medications, had wheat, dairy, or legume allergies or were vegetarian, were pregnant or lactating, had been diagnosed as diabetic or prediabetic, or had any musculoskeletal, cardiovascular, gastrointestinal, or immunological condition that could interfere with this study. 

### 2.3. Research Design

This study was a randomized clinical trial with 0 or 980 g of cooked whole green lentils seven days a week as a mid-day meal for 12 weeks. Testing for the human subject cohort took place in the Nutrition Research Laboratory at Montana State University. Participants reported to the lab for data collection over four visits. Subject consent and anthropometric measurements were completed at the first visit. Postprandial serum triglycerides were tested during visit 2 to ensure levels met inclusion criteria of ≥150 mg/dL (1.69 mmol/L). During visits 3 and 4, participants completed a high-fat meal challenge (50 g oral fat load) with blood collected at fasting and hourly timepoints for 5 h postprandially for blood marker assessment. Participants completed a 12-week dietary intervention between visits 3 and 4. See Supplementary Figure S1 for a summary of the study design.

### 2.4. Anthropometrics

All anthropometric measurements were collected from participants at baseline and after the 12-week intervention. Subjects were directed not to exercise, drink, or eat in the 3 h prior to testing. A validated [30] segmental multifrequency bioelectrical impedance analysis (SECA mBCA 515, Hamburg, Germany) was utilized to assess body mass index (BMI) and visceral adipose. Waist circumference was measured in triplicate using an anthropometric tape measure, with the average measurement used for analysis. Blood pressure was taken in the morning after subjects had been seated for at least 15 min. An automated measurement was taken in duplicate with the mean used for analysis.

### 2.5. Elevated Postprandial Triglyceride Screening

High-fat meal challenges containing 40 to 100 g of dietary fat are an established method to induce postprandial triglyceride and inflammation responses [25]. Participants were provided with a list of suggested breakfast and lunch meals containing approximately 50 g of fat. They were asked to consume a high-fat breakfast or high-fat lunch three to four hours before a visit with a single blood sample collection by a trained technician. Elevated postprandial triglycerides (ppTG) were defined as blood triglycerides greater than 150 mg/dL (1.69 mmol/L). Blood samples were run on-site using Picollo Xpress Chemistry Analyzer lipid panels (Abaxis, Union City, CA, USA). Participants who met ppTG criteria were enrolled into this study and continued to the dietary intervention.

### 2.6. Randomization

Block randomization was performed using the blockrand function in the R blockrand package (version 1.5) [31]. Fourteen blocks of six with two equally numbered levels were created, with the seed for each block individually set based on the last five integers returned via the sys.time (version 4.3.1) function. Participants were randomized to lentil (LEN) or control (CON) meal condition after confirmation of elevated ppTG to allow adequate time for preparation of their first week of study meals. Volunteers and clinical staff aiding in data collection during research visits were masked to treatment allocation. However, investigators performing research visits were aware of treatment allocation as they were the same individuals responsible for allocation of meals to participants for the duration of the study. Similarly, these investigators also analyzed the study data and were not masked. 

### 2.7. Lentil Sampling and Nutritional Analysis

Medium green lentils (CDC Richlea cultivar) used in the study diet were provided by AGT Foods Canada and sent to Medallion Labs (Minneapolis, MN, USA) for analytical testing. Functional properties and nutrient breakdown are provided in Supplementary Table S1. Dietary fiber was determined through the analytical method AOAC 991.43 [32].

### 2.8. Dietary Intervention

The 12-week dietary intervention consisted of 7 prepared mid-day meals (Table 1) each week that summed to either 0 g (CON) or 980 g (LEN) of cooked whole green lentils (provided by AGT Foods Regina, SK, Canada). This amount is equivalent to a daily dose of 140 g (0.7 cups) [33] of lentils which aligns with past studies reporting metabolic improvements from daily pulse consumption of 150 g (minimum–maximum: 54–360 g/day; cooked) [7]. 

Provided meals were prepared by a registered dietitian and professional chef on the research team. Meals were prepared in plastic to-go containers, labelled with heating instructions, and frozen for meal pick-up. Meal options included shepherd’s pie, soup, loaf with mashed potatoes, curry with basmati rice, street tacos, pasta with Bolognese sauce, and chili. CON and LEN meals were designed to have similar protein (g) and energy (kcals) content based on consumption of one of each meal per week with CON meals containing ground turkey or chicken instead of lentils. LEN meals had higher fiber and carbohydrate content, and less fat than CON meals. These compositional differences are representative of a diet substitution of lentils in lieu of meat (Supplementary Table S2). Because the dietary intervention followed a whole-foods approach, participants were able to identify that they were consuming either lentil-based meals or meat-based meals. However, participants were informed only that the purpose of this study was to study “how certain foods” influence the variables being assessed in the current study. Thus, while this study was not formally blinded so that a whole-foods approach could be utilized, participants were not aware that lentils were being compared to meals with animal protein. 

Participants were instructed to consume the provided meals during their regular lunch time—no specific time was required. Participants were asked to consume one of each of the seven meal options within their meal group the first week of the intervention. To increase satisfaction and diet adherence, participants were then allowed to select their meals based on preference for the remaining weeks. Other than consumption of the daily intervention meal, participants were instructed to maintain their normal dietary and physical activity patterns.

### 2.9. Diet Adherence

Adherence to daily consumption of intervention meals was monitored during meal pick-ups which occurred once per week or once every two weeks depending on participant preference. Researchers recorded type and number of meals provided to participants at every meal pick-up as well as any meals not consumed during the provided meal period. Failure to consume three or more meals in a one-week period resulted in a one-week extension of the intervention. In addition, participants could not miss any meals in the one-week period prior to their final visit. Any missed meals during the last week resulted in a one-week extension of the intervention period. To maintain equitable intervention time periods, participants were excluded from study analysis if adherence failures resulted in an intervention period exceeding 13 weeks.

### 2.10. Recent and Habitual Diet Surveys

Participants were also asked to provide information on recent and habitual dietary intake while seated during the high-fat meal challenge. Recent dietary intake was assessed through a 24 h dietary recall collected and analyzed with the Automated Self-Administered 24 h (ASA24) Dietary Assessment Tool, version (2020), developed by the National Cancer Institute, Bethesda, MD. The ASA24 was orally administered by research staff to participants. The generated report including portion sizes and specific food items consumed in the 24 h prior to the high-fat meal challenge to match food and beverage intake before repeat testing at the end of the nutrition intervention.

Habitual dietary intake was collected using the validated diet history questionnaire (DHQ III), offered by the National Cancer Institute. Participants completed the online survey using a lab-provided laptop. A 12-month recall was completed during visit 3 before the intervention and a 1-month recall was completed during visit 4 following the 12-week intervention. Participants were encouraged to give their best estimates of habitual intake during the recall with the acknowledgment that exact recollection of portion sizes for all food and beverage intake is unlikely. The DHQ III consists of 135 questions assessing food and beverage intake and 26 supplement questions assessing supplement intake. Questions address both dietary frequency and portion sizes. Healthy Eating Index (HEI, 2015) scores were obtained through DHQ III survey data output and included nine components based on adequacy (total fruit, whole fruit, total vegetables, greens and beans, whole grains, dairy, total protein foods, seafood and plant proteins, and fatty acids) and four components based on moderation (refined grains, sodium, added sugars, and saturated fats), as well as total HEI score (sum of the thirteen components). These scores are a measure of diet quality in compliance with the 2015–2020 US Dietary Guidelines for Americans [34,35]. In addition to HEI component scores, diet components expected to change with a lentil-based intervention were also analyzed as a dietary adherence check. These items included total fiber, g; insoluble fiber, g; and legumes, cups. Overall energy intake was also assessed.

### 2.11. Satiety and Gastrointestinal Symptom Surveys

To understand how participants respond to our lentil intervention, two surveys addressing satiety and gastrointestinal symptoms were delivered through email using REDCap (version 13.10.6) [28,29] on a randomly selected day, once a week for each week of the 12-week (or 13-week, if applicable) dietary intervention. At 4:00 p.m., participants were asked to rate their levels of hunger, fullness, satisfaction, desire to eat, and quantity of food they could eat in relation to the study meal they consumed mid-day. At 8:00 p.m., participants received a survey asking them to rank how they felt overall that day regarding common GI symptoms including abdominal discomfort, bloating, flatulence, and cramping. Survey questions and response scales are outlined in Supplementary Figure S2. Participant responses to both surveys were analyzed if they completed at least 8 of the 12 (or 13 if applicable) weekly surveys.

### 2.12. Blood Sampling

Participants were instructed not to participate in strenuous physical activity or consume alcohol in the 24 h prior to their visit and to complete a 10–12 h overnight fast before blood collection. Venous blood samples were collected via an intravenous catheter from the antecubital vein after a 3 mL waste withdrawal and then followed by a sterile saline flush. The fasting sample was drawn 20 min after catheter insertion. After fasting blood collection, participants consumed the high-fat meal followed by blood collection every hour for 5 h after meal ingestion. Blood was collected into serum separating and heparinized vacutainer tubes (BD Vacutainer, Franklin Lakes, NJ, USA). Serum tubes were allowed to clot for 15 min at room temperature before centrifugation (1200 RPM, 15 min). Serum aliquots were frozen at −80 °C until analysis. Whole blood from the heparinized tube was immediately utilized for blood marker analysis.

### 2.13. High-Fat Meal Challenge

The meal consisted of 58.3 g of salted butter (Tillamook, OR, USA) on three pieces of toasted whole wheat bread (Wheat Montana, Three Forks, MT, USA). The meal contained 50 g fat, 54 g carbohydrate, and 12 g protein with a total energy content of 714 kcal. Fat comprised approximately 43.1% of caloric content with 57% of total fat load from saturated fats. The meal contained approximately 9 g of dietary fiber. Participants were provided with water and caffeinated earl grey black tea (Bigelow, Fairfield, CT, USA) if they identified as habitual coffee consumers. Timing for the postprandial period began when participants started eating and they were given 15 min to consume the meal.

### 2.14. Analysis of Blood Markers

Blood markers were determined from whole blood collected in a sodium heparin tube run on Picollo Xpress Chemistry Analyzer lipid panels (Abaxis, Union City, CA, USA) which included total cholesterol (CHOL), high-density lipoprotein cholesterol (HDL), low-density lipoprotein cholesterol (LDL), triglycerides (TG), and glucose (GLU). Glycated hemoglobin was determined using the Affinion2 analyzer (Abbott, Princeton, NJ, USA) from whole blood collected in an EDTA additive tube. Serum insulin was determined through ELISA (MP Biomedicals, Irvine, CA, USA) performed according to manufacturer instructions, with the average used for analysis. Fasting blood GLU and insulin (INS) were used to determine the homeostatic model of insulin resistance (HOMA-IR), a measure of hepatic INS resistance at fasting calculated with the original HOMA-IR formula [36]. 

### 2.15. Analysis of Inflammation Biomarkers

Measurement of cytokine inflammation biomarkers was completed using high-sensitivity multiplexing technology (Bio-Rad Bio-Plex^®^ 200 HTS, Hercules, CA, USA) following procedures by Millipore (EMD Millipore Corporation, Billerica, MA, USA). Selected cytokines included interferon-gamma (IFN-γ), tumor necrosis factor alpha (TNF-α), granulocyte macrophage colony stimulating factor (GM-CSF), and five interleukins (IL) including IL-1β, IL-6, IL-10, IL-17, and IL-23. Plasma samples at each time point were run in duplicate and the mean was used for analysis. Sample values that were under the detectable limit were replaced with ½ the minimum detectable concentration for the respective cytokine for analysis [37].

### 2.16. Statistical Analysis

Analysis and visualizations were conducted in RStudio (2023.06.1) running R 4.3.1, with data visualized using ggplot2 (version 3.4.4) [38] and effects (version 4.2.2) [39] packages. Power for this study was calculated a priori using preliminary data for ppTG responses and 50% effect sizes consistent with published findings in animal models. Based on a two-sample *t*-test comparing pre- and post-intervention values, it was estimated that 18–24 participants would be required per group to achieve a power of 0.76 to 0.86 at α = 0.05.

Habitual dietary patterns, represented by DHQ-III HEI scores, were analyzed at baseline through general linear models to detect existing dietary differences between meal groups. Diet components expected to change with a lentil-based intervention, including total fiber g, insoluble fiber g, and legumes (cups), as well as total energy intake (kcals), were also assessed for baseline differences. To determine if intervention meal consumption changed participant dietary intake, linear mixed effects models were used to assess dietary components and HEI scores with an interaction predictor between meal and time (pre- or post-intervention) and a random effect for subject. Post-hoc multiple pairwise comparisons were performed to detect significant differences by group using Tukey’s HSD.

Linear mixed effects models were used to assess the impact of an interaction of time (week) and meal (LEN, CON) on satiety measures. Linear mixed effects models were used to assess GI symptom severity using symptom type and a week and meal interaction as predictors. Models for GI symptom severity and satiety included a random effect for subject.

General linear models were used to determine if physical and biological characteristics differed between meal groups before the intervention began. Descriptive summary statistics were performed to summarize participant characteristics as the average by meal group at baseline. General linear models were used to assess the impact of the dietary intervention on the following categories of variables: anthropometric measures (weight, fat mass percent, visceral adipose, and waist circumference); fasting blood lipid measures (CHOL, HDL, LDL, and TG); fasting glycemic measures (GLU, INS, and HOMA-IR); and fasting inflammation markers (GM-CSF, IFN-γ, TNF-α, IL-1β, IL-6, IL-10, IL-17, and IL-23). Fasting inflammation markers were normalized using R package bestNormalize (version 1.9.1) [40] prior to statistical analysis. Dependent variables were summarized as the change in values from baseline (post-intervention–pre-intervention). Predictor variables were common to each category of dependent variable as follows: anthropometric measures—meal group; blood lipid measures—meal group, visceral adipose tissue; glycemic measures—meal group, visceral adipose tissue, change in BMI, and HbA1c; inflammation measures—meal group, visceral adipose tissue. Predictor variables for glycemic measures were selected based on findings from Zeevi and colleagues [41]. Visceral adipose was included as a predictor variable in inflammation models because it represents a common difference between genders and is a key contributor to systemic inflammation levels [42]. It was also included in models examining blood lipid levels based on findings from Couillard and colleagues indicating its inclusion helps mitigate sex effects on blood lipid measures [43].

Analysis of postprandial metabolic (TG, GLU, and INS) and inflammatory (GM-CSF, IFN-γ, TNF-α, IL-1β, IL-6, IL-10, IL-17, and IL-23) measures were conducted by summarizing each makers’ measurements at timepoints 0 (fasting) and 1, 2, 3, 4, and 5 h postprandially as net area under the curve (AUC) using the auctime (version 2.0.0) [44] R package. Each dependent variable was then summarized as the change in AUC (post-intervention–pre-intervention). AUC values for inflammation markers were normalized using the R package bestNormalize (version 1.9.1) [40] prior to statistical analysis. General linear models were used to assess the impact of intervention meal group on each dependent variable. Predictor variables were identical to variables utilized in corresponding fasting models.

## 3. Results

### 3.1. General Characteristics of Participants

**Figure 1:**
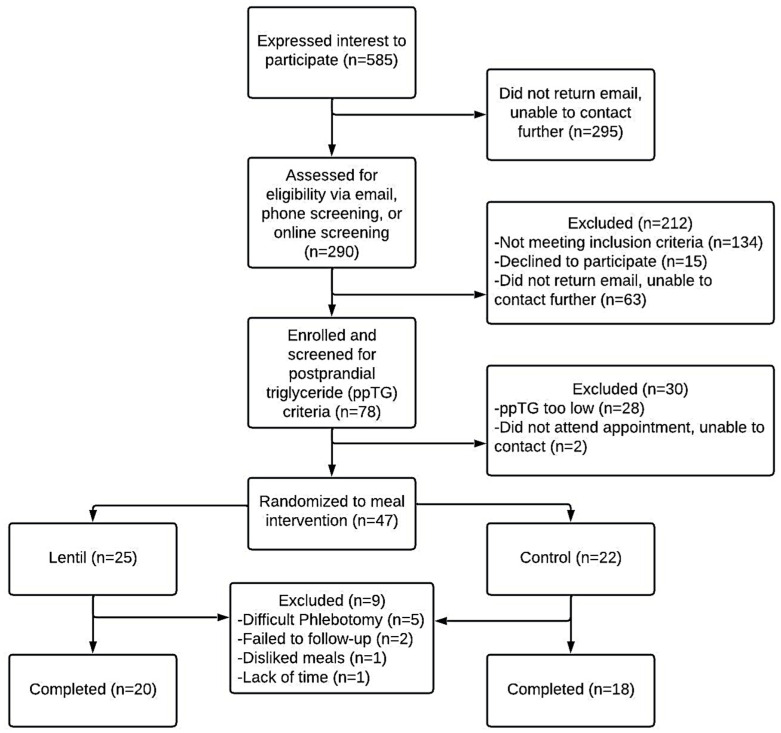
A CONSORT flowchart of the study design and enrollment. Flyers and emails were used to recruit interested individuals. Interested individuals contacted the research team, who sent additional information about this study. Two hundred and ninety individuals completed an initial eligibility screening with the research team. Forty-seven individuals met study requirements and were randomly allocated to CON or LEN experimental groups with varying weekly doses of lentils: CON, control (0 g/week) and LEN, lentil (980 g/week).

A total of 290 participants were screened for study participation of which 242 were excluded for not meeting inclusion criteria, lost to follow-up, or withdrew for non-intervention related reasons. Of the 78 individuals who completed screening for elevated postprandial triglycerides, 28 individuals (36%) were below the 1.69 mmol/L criteria, though all had met elevated waist circumference criteria. Forty-seven individuals were randomized to a meal intervention with a total of nine individuals lost for various reasons (Figure 1). Only one participant randomized to a diet withdrew from this study due to dislike of study meals. A total of 38 overweight and obese adults (mean ± SD); age (47.2 ± 13.1 years); BMI (34.4 ± 6.5 kg/m^2^) with elevated waist circumference (107.1 ± 13.9 cm females, 117.4 ± 0.7 cm males) completed the 12-week intervention. A total of 14 of the 38 participants completed an extra week of intervention meals (13 weeks total) due to reported missed meals in diet adherence checks.

Between meal groups at baseline, participants had similar anthropometric values as well as blood measures for HbA1c, GLU, HOMA-IR, TG, and blood pressure (Table 2). Fasting blood GLU and HbA1c were normal on average across meal groups and TG levels were optimal in the LEN group and intermediate in the CON group as compared to standard reference ranges. Fasting total CHOL and HDL cholesterol were different between groups at baseline, though CHOL values were optimal and HDL intermediate in both groups. The proportion of participants with metabolic syndrome was higher in the CON group compared to LEN with 61% and 20% meeting diagnostic criteria, respectively.

### 3.2. Anthropometric Measures

We did not observe meal group differences in the change in waist circumference (F = 0.2, *p* = 0.65), fat mass percentage (F = 1.13, *p* = 0.30), body weight (F = 0.06, *p* = 0.80), or visceral adipose tissue (F < 0.001, *p* = 0.98) over the 12-week intervention. Furthermore, these anthropometric metrics did not change in either meal group from pre- to post-intervention.

### 3.3. Habitual Diet Analysis

Participants had similar habitual diets at baseline as determined by meal group comparisons of several DHQ-III measures including the following: total and component HEI scores, total daily energy intake (kcals), legume consumption (cups), and total, soluble, and insoluble fiber consumption (grams). Of note, total fiber consumption averaged 17.3 g in the LEN group and 22.9 g in the CON group which both represent deficit intakes according to the 2020–2025 dietary guidelines for Americans [27]. In examining differences from the intervention, we found that LEN participants reported increased sodium intake over the intervention (t = 3.42, *p* < 0.01), and CON participants reported decreased dairy (t = 2.85, *p* = 0.04) intake and increased refined grain (t = 2.82, *p* = 0.04) intake. Table 3 summarizes HEI scores from pre- and post-intervention by group. 

As expected, participants in the LEN group had a significant increase in legume consumption (β = 0.53, *p* < 0.001) from a baseline daily average of 0.1 cups to 0.6 cups whereas average legume intake for CON remained unchanged at 0.2 cups. We also observed increases in the LEN group in the four HEI components that legume consumption is allocated to: total protein foods, seafood and plant proteins, total vegetables, and greens and beans. The LEN group also had an increase in total fiber (g) intake (β = 9.5, *p* < 0.01), insoluble fiber (g) intake (β = 6.6, *p* < 0.01), and soluble fiber (g) intake (β = 2.9, *p* = 0.01) over the course of the intervention whereas the CON group experienced a non-significant decrease in all fiber categories from baseline values (Supplementary Figure S3).

### 3.4. Satiety during the Intervention 

The overall response rate to the 4:00 p.m. survey on satiety was 90.2% (n = 35). The mean response rates were 89.6% and 90.8% for CON and LEN, respectively. Satiety measures were not different by meal group over the 12-week intervention (Supplementary Figure S4). Both groups self-reported a decrease in hunger (β = –0.07, *p* = 0.02) and desire to eat (β = 0.07, *p* = 0.02) over the course of the intervention period which corresponded to an increase in fullness (β = 0.09, *p* < 0.01). No change was observed in meal satisfaction over the 12 weeks with a mean score of 6.2 (CON) and 6.3 (LEN) out of 10. Only in the rating participants assigned to the amount of food they felt they could eat was a meal main effect (*p* = 0.04) observed with a lower average rating in the LEN (3.9) group than the CON (4.3) group. 

### 3.5. Gastrointestinal Symptoms during the Intervention

The overall mean response rate to the 8:00 p.m. survey on GI symptoms was 95.4% (n = 34). Mean response rates for the GI survey were 89.0% and 89.4%, respectively, for LEN and CON. There was no difference in symptom severity by meal group over the 12 weeks (*p* = 0.32). Symptom severity responses for both groups throughout the entire intervention were predominantly rated as none or mild (87.7%) with only 10% rated as moderate and 2.3% as severe. We observed a main effect for symptom type in our mixed effects modelling, with average reported flatulence as the GI symptom with the highest severity, though it was rated as mild throughout the duration of this study (*p* < 0.001). Flatulence was followed sequentially in symptom severity by bloating, abdominal discomfort, and cramping, with average ratings of 0.8, 0.68, 0.36, and 0.27, respectively, on a scale from 0 (none) to 4 (severe). Weekly fluctuations in symptom severity are visualized in Supplementary Figure S5.

### 3.6. Fasting Lipid and Glycemic Measures

We observed decreased cholesterol measures with regular lentil consumption (Table 4). Specifically, we detected differences between groups in fasting total CHOL (F = 8.64, *p* < 0.01) and LDL cholesterol (F = 5.53, *p* = 0.02) at the post-intervention time point. The LEN group averaged a 0.11 mmol/L decrease in total CHOL and a 0.03 mmol/L decrease in LDL whereas the CON group averaged a 0.36 mmol/L increase in total CHOL and a 0.29 mmol/L increase in LDL. We observed that fasting HDL differed by meal group (F = 14.08, *p* < 0.001) with LEN decreasing by 0.07 mmol/L and CON increasing by 0.11 mmol/L.

Participants entered the high-fat meal challenge with fasting TG concentrations considered normal (<1.7 mmol/L) to moderately elevated (2.0–5.6 mmol/L). We did not detect differences in fasting blood TG (F = 0.18, *p* = 0.68) between meal groups at the post-intervention time point.

Fasting GLU concentrations were normal (<5.5 mmol/L) prior to the high-fat meal challenge. Most participants maintained blood glucose levels under 7.8 mmol/L at the 2 h postprandial mark indicating a normal blood glucose response to a mixed meal, except for one participant who had a level of 7.88 mmol/L indicative of borderline prediabetes. We did not detect differences between meal groups in fasting blood GLU (F = 0.35, *p* = 0.56), fasting INS (F = 0.03, *p* = 0.85), HOMA-IR (F = 0.03, *p* = 0.87), or HbA1c (F = 0.34, *p* = 0.56). Changes to fasting blood markers from pre- to post-intervention are summarized in Table 4.

### 3.7. Postprandial Lipid Response

**Figure 2:**
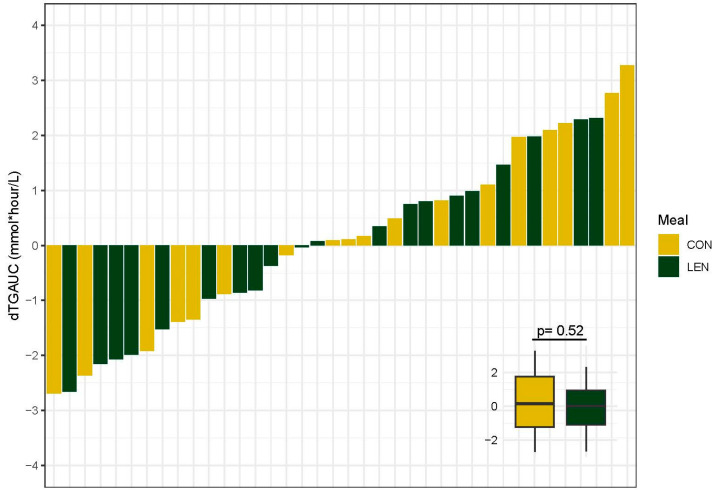
Summary plots of individual changes (post-intervention–pre-intervention) in triglyceride area under the curve (dTG_AUC_). dTG_AUC_ values represent the change in the sum of values from fasting and hourly timepoints for 5 h post high-fat meal ingestion. Each bar is representative of the value for one participant (n = 38). Difference in dTG_AUC_ between meal groups determined with ANOVA and displayed in inset. CON, control and LEN, lentil.

To better understand the impact of lentil consumption on postprandial metabolism, we examined postprandial lipid and glucose responses to a high-fat meal challenge. There was no effect of meal (F = 0.42, *p* = 0.52) on the change in TG AUC (dTG_AUC_) with an average dTG_AUC_ of 0.24 mmol/L for CON and −0.08 mmol/L for LEN. There was substantial variation between individuals in the dTG_AUC_ in both meal groups with responses ranging from a 2.7 mmol/L decrease to a 3.2 mmol/L increase from pre- to post-intervention. Variability in the change in TG AUC for each participant is summarized in Figure 2. During the high-fat meal challenge, 35 of the 38 participants reached the threshold of 1.98 mmol/L (175 mg/dL) for hypertriglyceridemia diagnosis in non-fasting states [45]. See Supplementary Figure S6 for a summary of the postprandial triglyceride response by meal group.

### 3.8. Postprandial Glycemic Response

**Figure 3:**
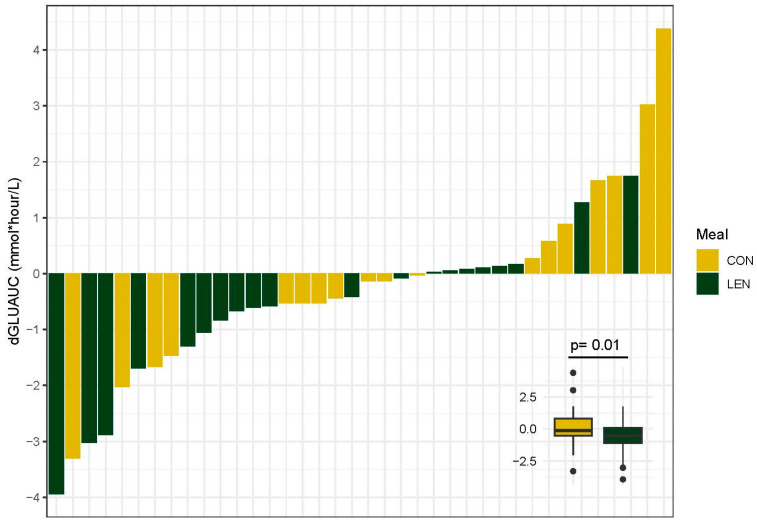
Summary plots of individual changes (post-intervention–pre-intervention) in glucose area under the curve (dGLU_AUC_). dGLU_AUC_ values represent the change in the sum of values from fasting and hourly timepoints for 5 h post high-fat meal ingestion. Each bar is representative of the value for one participant (n = 38). Difference in dGLU_AUC_ between meal groups determined with ANOVA and displayed in inset. CON, control and LEN, lentil.

We found that regular lentil consumption affected postprandial glucose but not insulin responses. Specifically, regular lentil consumption significantly lowered glucose AUC (GLU_AUC_) (β = −0.99, *p* = 0.01) with an average (mean) increase of 0.10 mmol/L in the CON group and a 0.67 mmol/L decrease in the LEN group, accounting for visceral adipose and change in BMI. The effect of intervention meal on the change in GLU_AUC_ (dGLU_AUC_) values for each participant is displayed in Figure 3. Lentil consumption did not impact insulin AUC (INS_AUC_) (F = 1.00, *p* = 0.32). Average glucose concentrations from the high-fat meal challenge before and after the intervention are displayed in Supplementary Figure S7.

### 3.9. Fasting and Postprandial Inflammation

**Figure 4:**
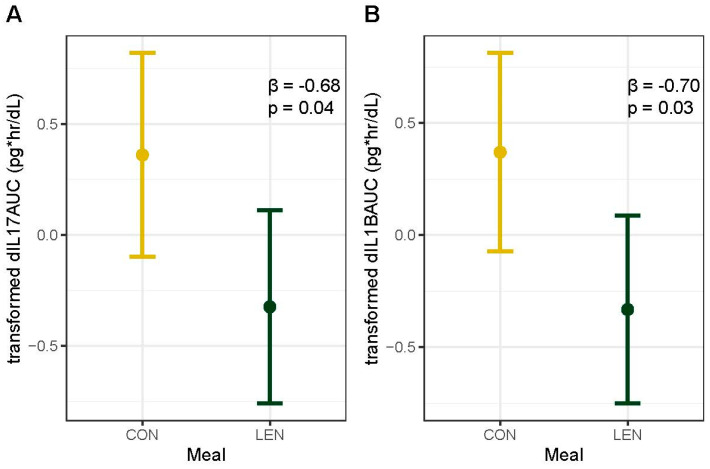
Main effect plot for meal group from (**A**) IL−17_AUC_ and (**B**) IL−1β_AUC_ linear models. Points indicate the average change in each marker as determined with the general linear model, and bars indicate 95% confidence intervals. Y-axis represents transformed values for each marker: orderNorm (IL−17_AUC_) and double reverse log (IL−1β_AUC_). β coefficient and *p*-value for LEN meal from model displayed in upper right corner. IL−1β, Interleukin 1 beta; IL−17, Interleukin 17; CON, control; and LEN, lentil.

As high-fat meals can induce an inflammatory response, we also examined a range of inflammatory markers. We found that regular lentil consumption decreased postprandial inflammation as measured with IL-1β_AUC_ (F = 5.4, *p* = 0.03) and IL-17_AUC_ (F = 4.80, *p* = 0.04) (Figure 4). Though not significant at α = 0.05, postprandial measures of Il-6, IL-10, TNF-α, GM-CSF, and IFNγ decreased in the LEN group and increased in the CON group from pre- to post-intervention, whereas both meal groups had a decrease in IL-23 (Supplementary Table S3). We did not detect a meal effect on changes in fasting inflammation markers, accounting for visceral adipose tissue (Supplementary Table S3). 

## 4. Discussion

Increased adiposity [2,3,4] and postprandial lipids [21,22,23,46] are established predictors of disease risk and are causally linked to low-grade inflammation and metabolic disease [25,26]. We recruited adults with high metabolic risk, marked by increased central adiposity and a high postprandial triglyceride response, and assessed the impact of regular lentil consumption compared to meat-based meals on fasting and postprandial lipid, glucose, and inflammatory measures. In addition, we surveyed participants about gastrointestinal (GI) symptoms throughout the 12-week intervention to assess the potential GI impacts of lentil consumption. Key findings of this study include decreased or maintained fasting cholesterol levels with lentil consumption and increased levels with consumption of meat-based meals. In addition, we observed improved postprandial glucose and inflammation responses to a high-fat meal challenge with long-term lentil consumption. Self-reported GI symptoms were not different between lentil and control groups, and all symptoms were reported as none or mild for the duration of the intervention. Importantly, metabolic improvements occurred independent of changes in anthropometric measures which suggests a direct impact of lentil consumption on metabolism.

In our study, fasting measures of lipid metabolism, including total and LDL cholesterol, were decreased with 12 weeks of daily lentil consumption. There are mixed results in the literature examining the impact of pulse consumption on fasting lipid markers. In a study examining the impact of eight weeks of mixed pulse (chickpeas, lentils, yellow split peas, and navy beans) intake at an average of 896 g/week on metabolism in adults with metabolic syndrome, no statistically significant changes were found in fasting triglyceride, total, or LDL cholesterol levels [47]. However, a study utilizing similar mixed pulses (lentils, chickpeas, peas, and beans) and an average weekly dose of 1050 g (dry weight) for eight weeks identified improvements to fasting cholesterol levels with pulse consumption in older adults [48]. Improvements to cholesterol with lentil consumption in our study may potentially be explained by the ability of fiber to bind bile acids, which decreases the return of bile acids to the liver and stimulates production of hepatic bile acids. Decreased hepatic cholesterol levels are replenished through cholesterol uptake from the blood which decreases serum cholesterol levels [49]. Lentils are also a rich source of saponins, bioactive compounds with a demonstrated ability to lower cholesterol levels via a variety of mechanisms including regulation of lipid metabolism and prevention of cholesterol absorption [50,51]. Habitual lentil consumption could also lower total saturated fat intake, a dietary component associated with cholesterol levels. However, this is not supported by our dietary survey data which indicate comparable saturated fat intake at pre- and post-intervention timepoints. Clinical trials investigating the impact of lentil consumption on lipid metabolism and bile acids are needed to provide more insight regarding the cholesterol lowering capability of lentils.

Long-term lentil consumption did not improve fasting markers of glycemic control including HbA1c and fasting glucose. There are mixed results in the literature concerning the impact of pulse consumption on glycemic control. Some randomized clinical trials found improvements to HbA1c and HOMA-IR with lentil consumption [9,20]. However, a recent meta-analysis of 28 long-term (3–72 weeks) randomized controlled trials examining the effects of pulse intake on glycemic control found that while long-term incorporation of pulses into dietary eating patterns attenuated fasting glucose in normoglycemic adults and lowered fasting glucose in participants with type 2 diabetes, consumption had no significant effect on fasting insulin, HOMA-IR, or HbA1c [8]. Importantly, only five studies examined the impact of single pulses and only three of these utilized the pulse in its whole food form. Because the metabolic benefits of pulse consumption vary by pulse variety, dosage, and duration of inclusion in the diet [15], more randomized controlled trials examining the impact of long-term consumption of single pulses on metabolic health are needed to clearly define their impact on glucose metabolism.

To better understand the impact of lentil consumption on postprandial metabolism, we examined postprandial glycemic and lipid responses to a high-fat meal challenge. We found that long-term, daily lentil consumption lowers postprandial glucose in a manner independent to the “same-meal” or acute effect of lentil consumption, as no lentils were consumed the day of the meal challenge. The mechanism of this effect is still debated, but lowered blood glucose with acute lentil ingestion has been associated with both fiber- and protein-dependent changes to glucose metabolism [52].While acute legume intake has been found to lower postprandial glucose levels [8,52], few studies have examined the impact of long-term pulse consumption on postprandial glucose [6]. A review [6] of randomized controlled trials (n = 18) investigating long-term legume consumption (>6 weeks) on markers of glycemic control identified only one study [53] examining postprandial glucose responses which found that 6 weeks of mixed bean consumption lowered 2 h postprandial glucose levels in individuals with diabetes. A number of factors impact the postprandial glycemic response including baseline glycemic status and insulin secretion [54]. However, we found no changes to fasting glycemic variables or postprandial insulin responses in response to long-term lentil consumption. Our results may be explained by short chain fatty acid (SCFA) production during bacterial fermentation of indigestible carbohydrates [55]. SCFA’s modulate metabolism in several ways including decreased hepatic glucose output [56]. This process modulates postprandial glycemia independent to mechanisms attributed to acute meal effects due to the delayed appearance of SCFA’s in the blood, secondary to meal ingestion and bacterial fermentation [57]. In this way, increased dietary fiber from lentil consumption may have reduced postprandial glucose concentrations. More studies are needed to decipher the relationship between long-term lentil intake and improved postprandial metabolism. This relationship is important to identify as long-term studies are a better indicator of the metabolic impacts of frequently applied dietary strategies to improve health.

Contrary to our hypothesis, fasting and postprandial triglyceride responses were not improved with 12 weeks of lentil consumption in our cohort of individuals with established elevated postprandial triglyceride responses. On average, we did observe a trend of small decreases in the lentil group as opposed to small increases in the meat-based control group. These results are in contrast to studies demonstrating modified postprandial lipemia responses with habitual diet changes [58] and studies demonstrating the ability of legume consumption to decrease blood triglyceride levels [9,59,60,61]. However, we are not aware of any studies specifically assessing long-term lentil intake on postprandial triglyceride responses. Our ability to detect changes with lentil consumption may have been masked by high interindividual variability in triglyceride responses both at baseline and after the intervention. Large interindividual variability in blood triglyceride responses were found in the large PREDICT 1 cohort in which factors such as gut microbiome composition influenced postprandial lipemia more than macronutrient composition of the meal [62]. In addition, lifestyle factors such as meal timing, physical activity [63], and sleep may also predict individual responses [62]. These results may support the use of personalized nutrition approaches in the application of any dietary strategy to improve metabolic health. 

We found that long-term consumption of lentil-based meals reduced postprandial IL-1β and IL-17 responses to a high-fat meal challenge compared to the meat-based meals. As IL-17 is a colonic inflammation marker, lowered IL-17 may be mediated by changes to the GI microbiome and microbial metabolites from habitual lentil consumption. Increased fiber consumption promotes the growth of microbiota-accessible carbohydrate degrading bacteria [64] and increases metabolic capacity to utilize carbohydrates by increasing carbohydrate active enzymes [65]. These changes promote microbial production of fiber fermentation derivatives such as SCFAs. SCFAs beneficially influence gut health in many ways including reducing colonic inflammation and improving gut epithelial barrier integrity. Anti-inflammatory effects of SCFAs include enhancing activity of immunosuppressive regulatory T-cells [66] and inhibiting proinflammatory cytokine production [67,68]. A lentil supplementation study in the murine model identified decreased colonic proinflammatory cytokines and increased expression of the tight junction protein occludin [69], which suggests lentils may confer a protective effect against colonic inflammation through improved barrier function. Though IL-1β is not an intestinal inflammation marker, previous work has shown that postprandial increases in IL-1β secretion via peritoneal macrophages occur in a glucose dependent manner [70]. It is therefore possible that the decrease in IL-1β in the lentil group may be a reflection of their lower postprandial glucose responses. We did not observe changes in fasting markers between meal groups which echoes previous findings in eight-week legume feeding trials in humans [71,72]. Further research on postprandial inflammation may consider the inclusion of gastrointestinal health markers and relationships with glucose metabolism. 

Given the impacts of lentils on GI health, it is important to note that microbial responses to fiber depend on the fiber type and the host GI microbiome [73]. The broad fiber analysis of our study lentils limits our ability to speculate based on fiber type, but we might reasonably expect a fiber composition of 3% oligosaccharides (dry weight), 44% total starch content, and 11% of total starch as resistant starch based on chemical analysis of other green lentil cultivars [74]. Resistant starch displays low-to-intermediate specificity, meaning it can be metabolized by many bacteria. As substrate usage is not taxa-specific, individuals display high interindividual variability in microbial responses to low-specificity fiber types based on their initial GI communities [73]. Microbiome response variability may also contribute to variable immunological profiles after fiber consumption [65]. Thus, it is possible that our participant microbiomes had personalized compositional and metabolic responses to fiber consumption which contributed to the observed postprandial responses in this study. Future studies assessing the impact of lentil consumption on inflammation should consider the contributions from microbial fermentation, with particular care given to the fermentability of fiber types and fermentation indicators such as fecal pH or serum SCFA.

Importantly, improvements in metabolism with the study dose of 980 g of lentils per week were not associated with increased GI symptoms as compared to control meals. This finding is impactful as GI symptoms are infrequently reported in pulse interventions, and we are aware of only one study exclusively examining lentils that reported symptomology, which was a study conducted by our research group [20]. Additionally, legume consumption by US adults is low, with an estimated 15% of the population consuming legumes at the amount (≥87.5 g/d) recommended to achieve disease prevention benefits [75]. Consumers commonly report GI symptoms as a barrier to increased consumption [18]. In this study, the GI symptom with the highest average severity was flatulence; however, severity was still reported as mild throughout the duration of the intervention. Furthermore, severity was not different between meal groups. GI discomfort largely arises from bacterial fermentation of indigestible complex carbohydrates such as soluble fiber and resistant starch [76,77,78]. Lentils are primarily rich in insoluble fiber [79] but are also high in starch content (~36 g/100 g) (Supplementary Table S1). The analysis performed on the lentils used in this study did not include further characterization of starch type, so it is unclear if the lack of GI discomfort with lentil intake was due to inadequate substrate for increased bacterial fermentation and secondary gas production or substrate adaptation. However, because our intervention dose was over the current USDA recommendation for pulse consumption, we have effectively demonstrated that USDA pulse consumption recommendations may be met with lentil intake without an increase in gastrointestinal symptoms. 

We recognize there are additional factors and limitations which may have influenced changes to metabolic and inflammatory markers that were not measured in the current study. First, the interpretation of study results is constrained to individuals who are representative of the study sample. This sample comprises individuals at an increased metabolic risk, as indicated by elevated waist circumference and postprandial triglyceride levels. In addition, while intervention meals were matched for protein and energy content, the carbohydrate, fiber, and fat content were different between meals. This variance in macronutrient composition represents the difference between lentil-based meals and meat-based meals and is the basis of our study design and statistical analysis. However, we cannot exclude the possibility that these differences impacted outcome measures in a manner independent to lentil consumption. Although participants were asked to maintain their regular dietary and physical activity patterns for the duration of this study, it is possible that alterations to eating and exercise patterns during the 12-week period may have impacted study measures. However, as anthropometric measures did not change between groups during this study, large lifestyle shifts do not seem likely. In addition, total HEI scores were not different for either meal group from pre- to post-intervention, indicating a relatively stable diet intake. The decrease in dairy intake and increase in refined grain intake in the control group as well as the increase in sodium intake in the lentil group may be due to changes in diet in response to the intervention meal. For example, single-serving yoghurt and cheese are commonly consumed lunch foodstuffs which would be replaced by the intervention meals which were all low in dairy content. The intervention meals were also low in refined grain and sodium content, so the increase in these scores may be reflective of compositional changes to the next meal consumed. It is also possible that changes to HEI component scores are reflective of estimation errors common to all self-reported dietary intake questionnaires. There is some evidence to indicate that sex differences are an important variable to consider regarding fasting and postprandial lipid and glycemic measures [62], but we were unable to do so with the limited sample size of male participants. Our male participants were not detected as outliers in models examining metabolic and inflammatory changes. The survey used to collect GI symptom and severity data was self-reported. This method has been used and reported as an effective measure of gastrointestinal symptoms in various studies [80,81,82]. However, the survey results could be further validated with external observations and ratings by trained medical professionals. Lastly, lentils are a rich source of dietary bioactives which represent an additional mechanism for improvement to metabolic health measures. However, these nutrients were not analyzed so we cannot define their impact on study results. 

In an effort to comprehensively measure the impact of our lentil dose on metabolic health, we measured postprandial lipid, glucose, and inflammation responses in addition to corresponding fasting metrics. While the importance of postprandial measures in relation to metabolic health is well-established, these measures are not consistently measured in pulse intervention studies—particularly in non-acute intervention studies. The completion of these measures in our study more comprehensively characterized the impact of long-term lentil intake on metabolic health. Other strengths of this study include the use of a whole-food based intervention in free-living adults with meals matched in protein and calorie content. This approach is most representative of dietary changes likely to be made by individuals wanting to incorporate more pulses into their diet. In addition, only one pulse, lentils, were tested as opposed to studies examining increased pulse intake via incorporation of multiple pulses. Because the impact of pulse intake on metabolic health is both dose and pulse-specific, randomized controlled trials examining individual pulses are necessary to fully characterize their impact on metabolic health and establish dietary recommendations. Lastly, the dietary dose of 980 g/week meets, and exceeds, the recommendation set for pulse intake by the USDA and is reflective of long-term lentil intake with a 12-week intervention period—criteria which are less frequently examined in pulse intervention studies. In this manner, our study results are highly applicable towards the development of dietary recommendations for lentil consumption to improve metabolic health. Gastrointestinal (GI) symptoms associated with pulse intake are also infrequently measured in the literature. Our study directly contributes to this knowledge gap by collecting questionnaires from participants on a weekly basis during the intervention period regarding commonly reported GI symptoms associated with the intervention meals. 

## 5. Conclusions

In conclusion, our findings provide evidence that long-term lentil consumption in individuals with increased risk for metabolic disease, as defined by elevated waist circumference and postprandial triglyceride responses, can mitigate increases in fasting cholesterol levels as well as postprandial glucose and inflammatory responses. Our findings help address the current limited body of research regarding the impact of legume consumption on gastrointestinal symptomology with the important finding that lentil consumption over the USDA recommended dose is not associated with increased GI distress. These results implicate daily consumption of lentils as a safe and effective dietary strategy to improve metabolic health which may help reduce barriers to increased consumption for health benefit. This information further informs the development of pulse-based dietary strategies to lower disease risk and to slow or reverse metabolic disease progression in at-risk populations. Further investigation of the long-term impact of other pulse crops on fasting and postprandial metabolism and systemic inflammation is warranted.

## Figures and Tables

**Table 1:** Overview of intervention meal composition by group.

Meal	CON—0 g/week	LEN—980 g/week
Bolognese	Turkey Bolognese + ½ cup cooked rotini pasta	Lentil Bolognese + ½ cup cooked rotini pasta
Curry	Chicken curry + 1/3 cup cooked basmati rice	Lentil curry + 1/3 cup cooked basmati rice
Loaf	Turkey loaf + ¼ cup mashed potatoes + ½ cup cooked zucchini	Lentil loaf + ¼ cup mashed potatoes + ½ cup cooked zucchini
Taco	Turkey taco filling + ½ oz shredded cheddar cheese + 1 Tbsp salsa + 1 Tbsp sour cream + 2 street flour tortillas	Lentil taco filling + ½ oz shredded cheddar cheese + 1 Tbsp salsa + 1 Tbsp sour cream + 2 street flour tortillas
Soup	Chicken soup + 3 packets saltine crackers	Lentil soup
Shepherd’s Pie	Chicken shepherd’s pie + 2 dinner rolls + 1 pat butter	Lentil shepherd’s pie + 1 dinner roll + 2 pats butter
Chili	Turkey chili + 3 packets saltine crackers	Lentil chili + 1 packet saltine crackers

**Table 2:** Participant characteristics at baseline, grouped by assigned dietary intervention (*n* = 38). Blood profile determined from fasting venous blood sample. Bold *p*-values indicate strong evidence of a difference between meal groups.

	CON (*n* = 18)	LEN (*n* = 20)	*p*-Value
Age (years)	43.2 (14.0)	50.6 (11.5)	0.08
Sex (M/F)	1/17	2/18	0.58
BMI (kg/m^2^)	35.9 (7.6)	33.1 (5.1)	0.19
Fat mass (%)	45.1 (6.2)	44.0 (6.1)	0.57
Visceral adipose (L)	3.6 (2.1)	3.1 (1.8)	0.45
MetS Presence (Y/N) ^1^	11/7	4/16	**<0.01**
HbA1c (%)	5.4 (0.3)	5.5 (0.2)	0.48
Fasting Glucose (mmol/L)	5.5 (0.5)	5.4 (0.3)	0.43
HOMA-IR	3.8 (2.6)	4.2 (8.2)	0.86
Total Cholesterol (mmol/L)	4.4 (0.7)	5.1 (0.7)	**0.01**
HDL Cholesterol (mmol/L)	1.2 (0.2)	1.5 (0.4)	**0.01**
LDL Cholesterol (mmol/L)	2.85 (0.64)	3.28 (0.64)	0.05
Triglycerides (mmol/L)	1.7 (0.8)	1.6 (0.7)	0.52
Blood Pressure (mmHg)			
Systolic	117.8 (11.9)	111.2 (11.4)	0.09
Diastolic	82.6 (10.8)	76.5 (9.4)	0.08

^1^ Criteria were based on the National Cholesterol Education Program Adult Treatment Panel III definition of metabolic syndrome which includes criteria on central obesity, hypertension, insulin resistance, and dyslipidemia. Data represent mean (standard deviation). Sex and MetS Presence *p*-values determined through 2-sample test for given proportions; all other *p*-values determined with ANOVA. Abbreviations: control, CON; lentil, LEN; body mass index, BMI; Metabolic Syndrome, MetS; hemoglobin A1c, HbA1c; homeostatic model of insulin resistance, HOMA-IR; high-density lipoprotein, HDL; low-density lipoprotein, LDL.

**Table 3:** Habitual dietary intake as depicted by HEI total and component scores before and after the 12-week dietary intervention. HEI component scores were calculated through an online DHQ III in which participants self-reported the types and frequency of foods consumed.

Component	Maximum Points	Score			
		CON (*n* = 18)		LEN (*n* = 20)	
		Pre	Post	Pre	Post
Adequacy					
Total fruits	5	3.6 ± 1.4	2.8 ± 1.5	3.3 ± 1.6	3.2 ± 1.6
Whole fruits	5	4.4 ± 1.0	3.9 ± 1.5	4.0 ± 1.5	3.9 ± 1.6
Total vegetables	5	4.0 ± 1.3	4.1 ± 1.0	3.9 ± 1.3	4.5 ± 1.0 *
Greens and beans	5	4.3 ± 1.2	4.2 ± 1.4	4.0 ± 1.5	4.8 ± 0.8
Whole grains	10	2.7 ± 1.1	3.3 ± 1.5	3.3 ± 1.6	2.9 ± 2.2
Dairy	10	7.4 ± 2.0	6.3 ± 2.0 *	6.8 ± 2.1	5.9 ± 2.5
Total protein foods	5	4.9 ± 0.4	4.9 ± 0.2	4.7 ± 0.9	5.0 ± 0.2
Seafood and plant proteins	5	4.4 ± 1.0	4.7 ± 0.7	4.2 ± 1.1	4.8 ± 0.8
Fatty acids	10	3.7 ± 2.4	4.1 ± 2.2	4.9 ± 2.5	5.0 ± 2.5
Moderation					
Refined grains	10	9.1 ± 1.4	7.1 ± 2.4 *	8.5 ± 2.2	8.2 ± 2.7
Sodium	10	3.4 ± 3.3	1.9 ± 1.8	4.1 ± 2.7	2.2 ± 2.1 *
Added sugars	10	8.0 ± 2.3	8.5 ± 1.6	7.9 ± 2.5	8.7 ± 1.5
Saturated fats	10	3.3 ± 2.9	4.0 ± 2.0	4.7 ± 2.9	5.5 ± 2.8
Total	100	63.1 ± 8.1	59.6 ± 7.7	64.1 ± 10.8	64.4 ± 7.5

Data represent mean ± sd. Linear mixed modeling with Tukey’s HSD used to determine differences between group means. Difference (*p* < 0.05) between pre and post scores identified with *. Abbreviations: LEN, lentil; CON, control; and DHQ, diet history questionnaire.

**Table 4:** Change in fasting blood markers (post-intervention–pre-intervention), grouped by dietary condition. Blood profile determined from fasting venous blood sample. Bold *p*-values indicate strong evidence of a difference between meal groups.

	CON (n = 18)	LEN (n = 20)	*p*-Value
∆ Total Cholesterol (mmol/L)	0.36 (0.43)	−0.11 (0.50)	**<0.01**
∆ HDL Cholesterol (mmol/L)	0.10 (0.14)	−0.07 (0.15)	**<0.001**
∆ LDL Cholesterol (mmol/L)	0.30 (0.36)	−0.03 (0.43)	**0.02**
∆ Triglycerides (mmol/L)	−0.21 (0.54)	−0.06 (0.73)	0.51
∆ GLU (mmol/L)	−0.05 (0.44)	−0.06 (0.34)	0.56
∆ INS (mmol/L)	−2.40 (8.84)	−3.83 (22.35)	0.85
∆ HbA1c (%)	0.06 (0.38)	0.03 (0.17)	0.56
∆ HOMA-IR	−0.59 (2.29)	−0.95 (5.23)	0.87

Data represent mean change (∆) in fasting marker (post-intervention–pre-intervention) (standard deviation). *p*-values determined with ANOVA. Abbreviations: control, CON; lentil, LEN; high density lipoprotein, HDL; low density lipoprotein, LDL; glucose, GLU; insulin, INS; hemoglobin A1c, HbA1c; and homeostatic model of insulin resistance, HOMA-IR.

## Data Availability

The data presented in this study are available on request from the corresponding author. The data are not publicly available due to participant confidentiality.

## Supplementary Materials

The following supporting information can be downloaded at: https://www.mdpi.com/article/10.3390/nu16030419/s1. Supplementary Figure S1. Timeline of study events. Baseline anthropometric and screening questionnaires completed at visit 1 followed by testing for elevated postprandial triglycerides (>1.98 mmol/L) at visit 2. Individuals with elevated postprandial triglycerides were enrolled in this study and completed a 5 h high-fat meal challenge, dietary questionnaires, and stool collection at visit 3. Participants were randomly allocated to CON or LEN experimental groups with varying weekly doses of lentils, CON, control (0 g/week) and LEN, lentil (980 g/week), for 12 weeks. Repeat testing of visit 3 metrics and anthropometric testing was completed at visit 4. Supplementary Figure S2. Survey questions assessing satiety and gastrointestinal issues. Online surveys were delivered electronically once a week each week of the 12-week dietary intervention. Satiety was scored on a scale from 0 to 10 as indicated below. Gastrointestinal symptom severity was scored as follows: 0 = None, 1 = Mild, 2 = Moderate, and 3 = Severe. Supplementary Figure S3. Differences in daily fiber intake from pre- to post-intervention by meal group. Significance determined using paired *t*-test with * indicating significant effect of time (pre- to post-intervention) at a level of α = 0.05. Supplementary Figure S4. Self-reported satiety measures over the 12-week intervention by meal group. An online satiety survey was electronically delivered at 4 p.m. once a week for each week of the 12-week dietary intervention. Participants were asked to self-report hunger, fullness, satisfaction, desire to eat, and amount of food that could be eaten (see Supplementary Figure S2 for survey questions). All measures on 10-point scale. Points indicate group means and bars represent ± standard error. LEN, lentil and CON, control. Supplementary Figure S5. Self-reported gastrointestinal symptoms over the 12-week intervention by meal group. An online survey addressing gastrointestinal symptoms was electronically delivered at 8 p.m. once a week for each week of the 12-week dietary intervention. Participants were asked to self-report symptoms of flatulence, bloating, cramping, and abdominal discomfort (see Supplementary Figure S2 for survey questions). All measures on 4-point scale (0 = none, 1 = mild, 2 = moderate, and 3 = severe). Points indicate group means and bars represent ± standard error. LEN, lentil and CON, control. Supplementary Figure S6. Postprandial triglyceride (TG) response to a 50 g, high-fat meal challenge by meal group before and after the 12-week dietary intervention. Points indicate group means and bars represent ± standard error. LEN, lentil and CON, control. Supplementary Figure S7. Postprandial glucose (GLU) response to a 50 g, high-fat meal challenge by meal group before and after the 12-week dietary intervention. Points indicate group means and bars represent ± standard error. LEN, lentil and CON, control. Supplementary Table S1. Nutrient and functional analysis of whole green lentils used in the 12-week intervention. Supplementary Table S2. Overview of intervention meals and their nutrient profile. Profile based off the first week of study meals, where participants received one of each of the seven study meals. Macronutrient totals were rounded to nearest whole number. Supplementary Table S3. Change in fasting and postprandial inflammation by meal group before and after the 12-week dietary intervention. Fasting values represent timepoint 0 (fasting) collected prior to 50 g, high-fat meal challenge. Postprandial values summarized as net area under the curve (AUC) using fasting and 1-, 2-, 3-, 4-, and 5-h postprandial measurements. Delta values calculated as (post-intervention–pre-intervention) and expressed by Δ Baseline to represent changes in fasting inflammation and Δ AUC to represent changes in postprandial inflammation. Values as mean (standard error). Bold *p*-values indicate strong evidence of a difference between meal groups.
