# Supplementary material for: Twelve Weeks of Daily Lentil Consumption Improves Fasting Cholesterol and Postprandial Glucose and Inflammatory Responses—A Randomized Clinical Trial
Source: Nutrients. 2024 Jan 31;16(3):419. doi: 10.3390/nu16030419 (PMC10857178; doi:10.3390/nu16030419)
Supplement: Supplementary file 1 [file nutrients-16-00419-s001.zip › nutrients-2837217-supplementary.pdf]

Supplementary Material

Title: Twelve Weeks of Daily Lentil Consumption Improves Fasting Cholesterol and Postprandial Glucose and Inflammatory Responses—A Randomized Clinical Trial.

Authors: Morgan L. Chamberlin[1], Stephanie M.G. Wilson[2,3], Marcy E. Gaston[4], Wan-Yuan Kuo[1], Mary P. Miles[1*]

[1] Department of Food Systems, Nutrition, and Kinesiology, Montana State University, Bozeman, MT 59717, USA; morgan.chamberlin@student.montana.edu (M.L.C.); wanyuan.kuo@montana.edu (W.-Y.K.)

[2] United States Department of Agriculture, Agricultural Research Service Western Human Nutrition Re-search Center, Davis, CA 95616, USA; smgwilson@ucdavis.edu

[3] Texas A&M, Institute for Advancing Health Through Agriculture, College Station, TX 77845, USA

[4] Department of Human Ecology, SUNY Oneonta, Oneonta, NY 13820, USA; mar-cy.gaston@oneonta.edu

* Correspondence: mmiles@montana.edu; Tel.: +1-406-994-6678

1 Supplementary Data

No supplementary data is provided.

2 Supplementary Figures and Tables

2. Supplementary Figures and Tables

Supplementary Figure S1. Timeline of study events. Baseline anthropometric and screening questionnaires completed at Visit 1 followed by testing for elevated postprandial triglycerides (>1.98 mmol/L) at Visit 2. Individuals with elevated postprandial triglycerides were enrolled in the study and completed a 5-hour high-fat meal challenge, dietary questionnaires, and stool collection at Visit 3. Participants were randomly allocated to CON or LEN experimental groups with varying weekly doses of lentils: CON, control (0 g/week); LEN, lentil (980 g/week) for 12 weeks. Repeat testing of Visit 3 metrics and anthropometric testing was completed at Visit 4.

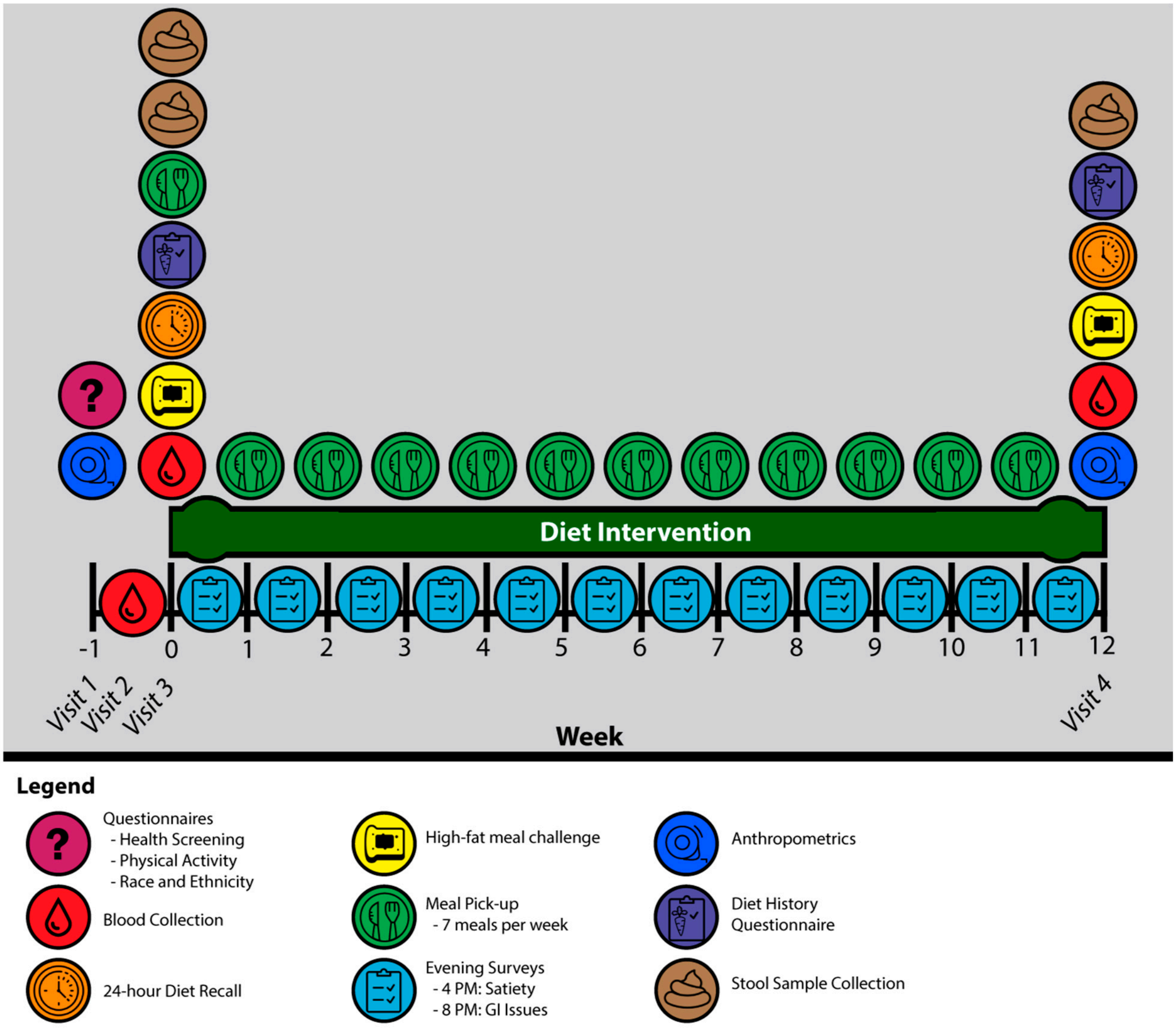

Supplementary Figure S2. Survey questions assessing satiety and gastrointestinal issues. Online surveys were delivered electronically once a week each week of the 12-week dietary intervention. Satiety was scored on a scale from 0 – 10 as indicated below. Gastrointestinal symptom severity was scored as follows: 0 = None, 1 = Mild, 2 = Moderate, 3 = Severe.

**Question**	**Response**
4:00 PM Satiety	Ordinal
How hungry are you?	0 = not at all, 10 = extremely
How full are you?	0 = not at all, 10 = extremely
How satisfied do you feel?	0 = not at all, 10 = extremely
How strong is your desire to eat?	0 = very weak, 10 = very strong
How much do you think you could (or would want to) eat right now?	0 = nothing, 10 = A lot
8:00 PM Gastrointestinal Issues	Ordinal
Please rate below how on how you feel OVERALL today.	
Flatulence (having excessive stomach or intestinal gas)	None, Mild, Moderate, Severe
Bloating (the abdomen feels full and tight, often interpreted as excessive intestinal gas)	None, Mild, Moderate, Severe
Cramping (a type of pain that comes and goes. Can be very uncomfortable but is relieved by passing gas or having a bowel movement	None, Mild, Moderate, Severe
Abdominal Discomfort (a feeling of discomfort or pain located in the upper or lower abdominal area)	None, Mild, Moderate, Severe

Supplementary Figure S3. Differences in daily fiber intake from pre- to post- intervention by meal group. Significance determined using paired t-test with * indicating significant effect of time (pre- to post- intervention) at a level of α=0.05.

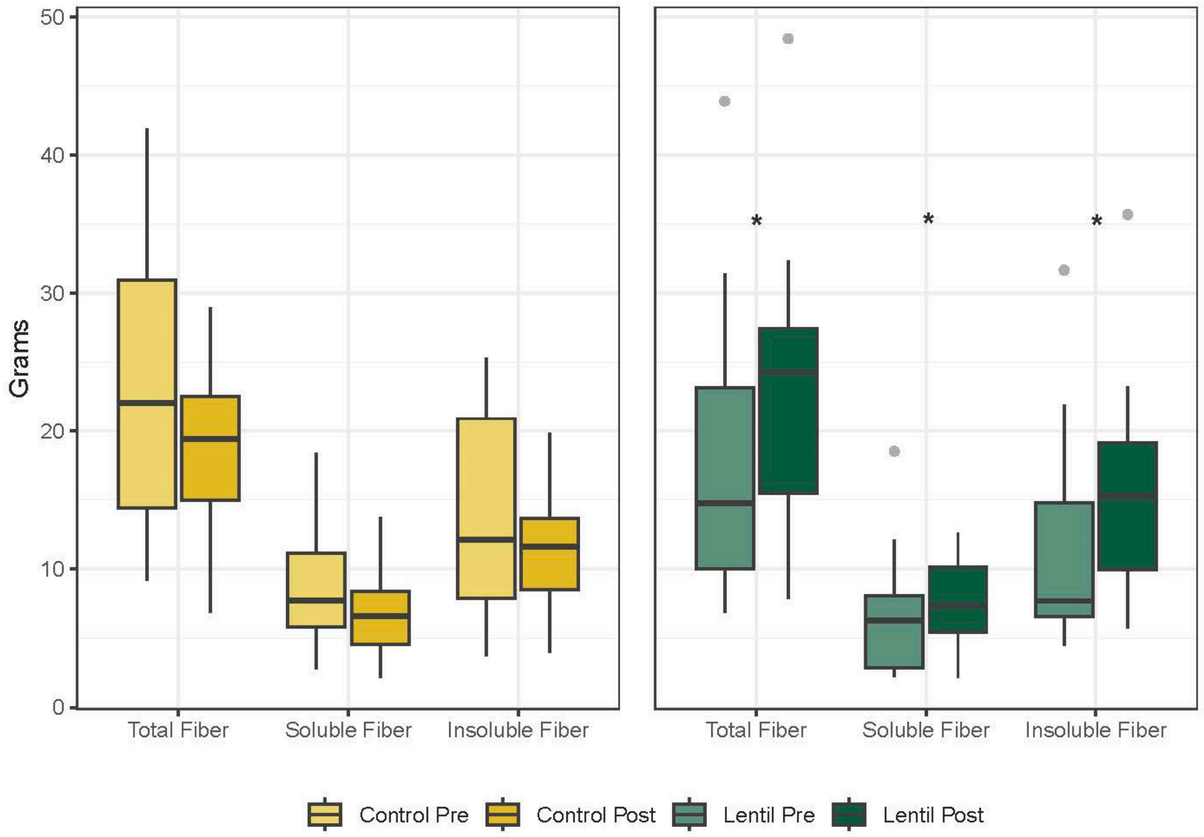

Supplementary Figure S4. Self-reported satiety measures over the 12-week intervention by meal group. An online satiety survey was electronically delivered at 4 pm once a week for each week of the 12-week dietary intervention. Participants were asked to self-report hunger, fullness, satisfaction, desire to eat, and amount of food that could be eaten (see Supplemental Figure 2 for survey questions). All measures on 10-point scale. Points indicate group means, bars represent ± standard error. LEN, lentil; CON, control.

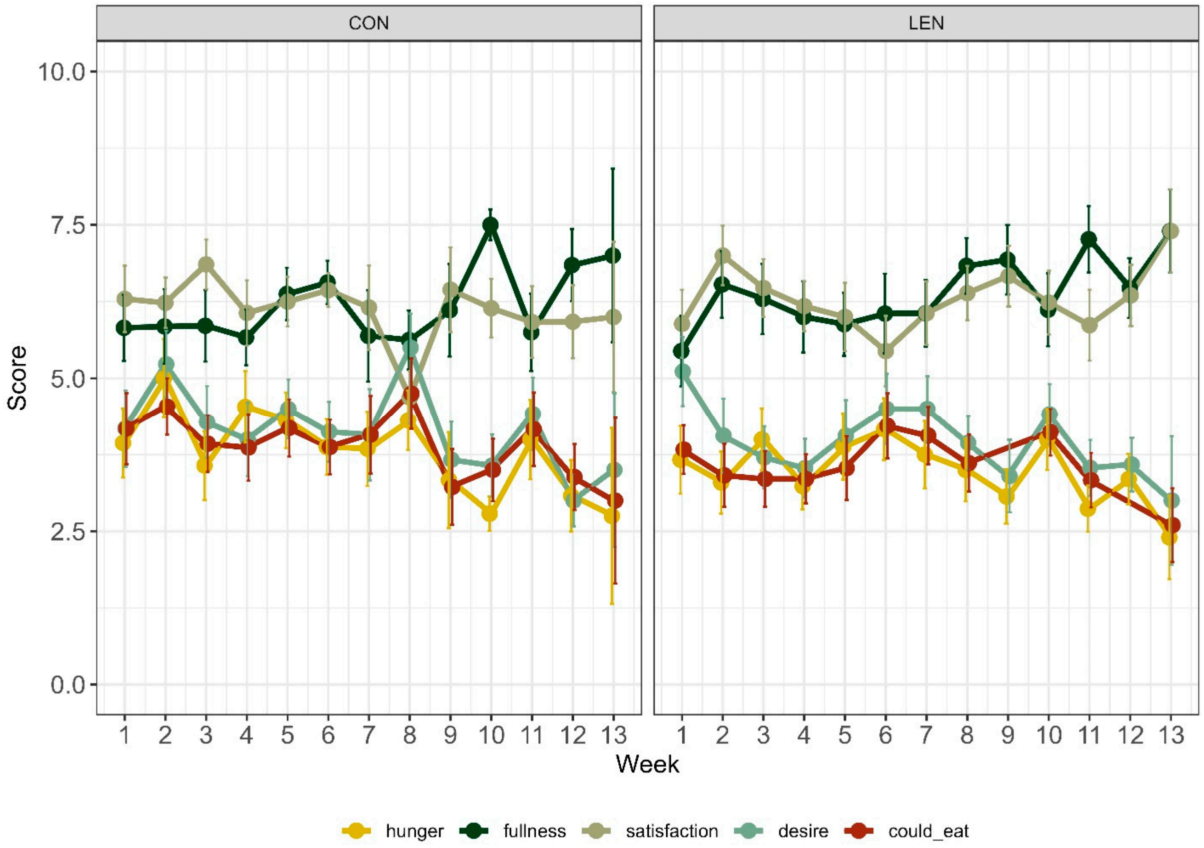

Supplementary Figure S5. Self-reported gastrointestinal symptoms over the 12-week intervention by meal group. An online survey addressing gastrointestinal symptoms was electronically delivered at 8 pm once a week for each week of the 12-week dietary intervention. Participants were asked to self-report symptoms of flatulence, bloating, cramping, and abdominal discomfort (see Supplemental Figure 2 for survey questions). All measures on 4-point scale (0 = none, 1 = mild, 2 = moderate, 3 = severe). Points indicate group means, bars represent ± standard error. LEN, lentil; CON, control.

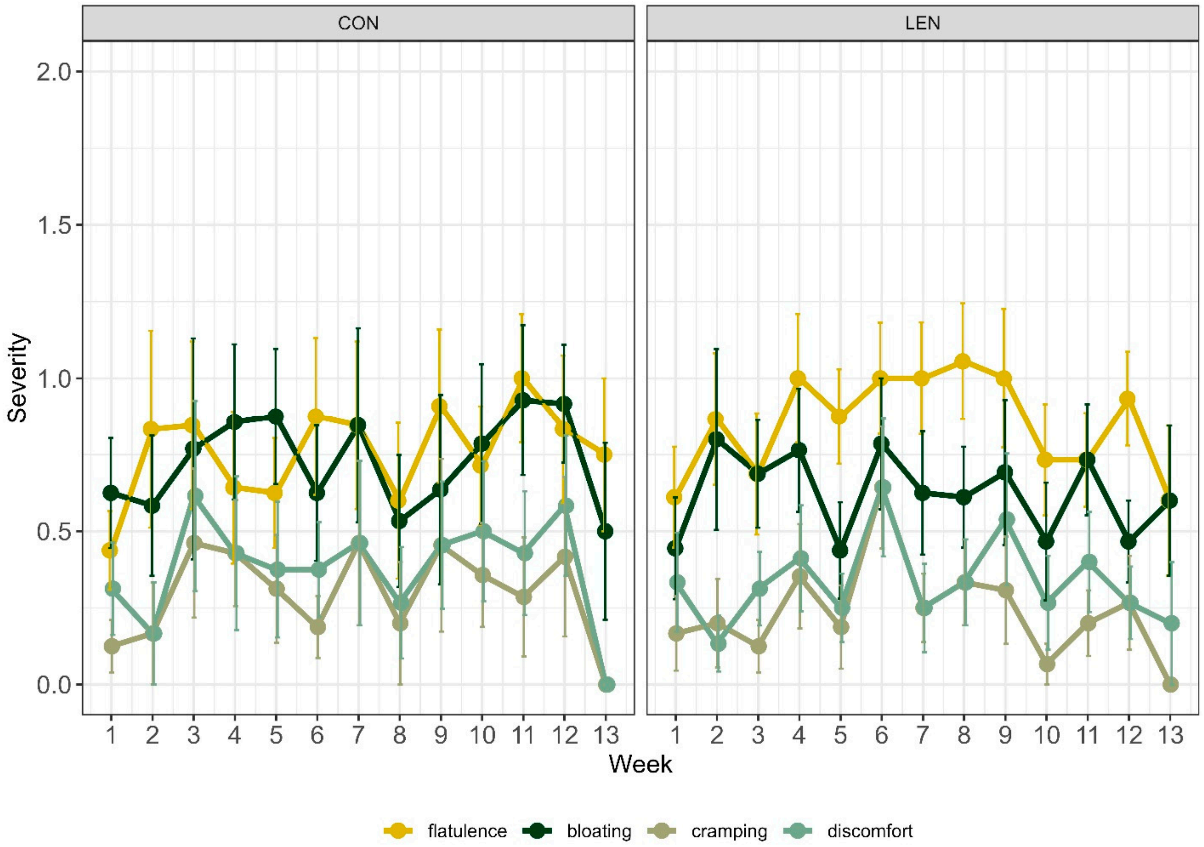

Supplementary Figure S6. Postprandial triglyceride (TG) response to a 50-gram, high-fat meal challenge by meal group before and after the 12-week dietary intervention. Points indicate group means, bars represent ± standard error. LEN, lentil; CON, control.

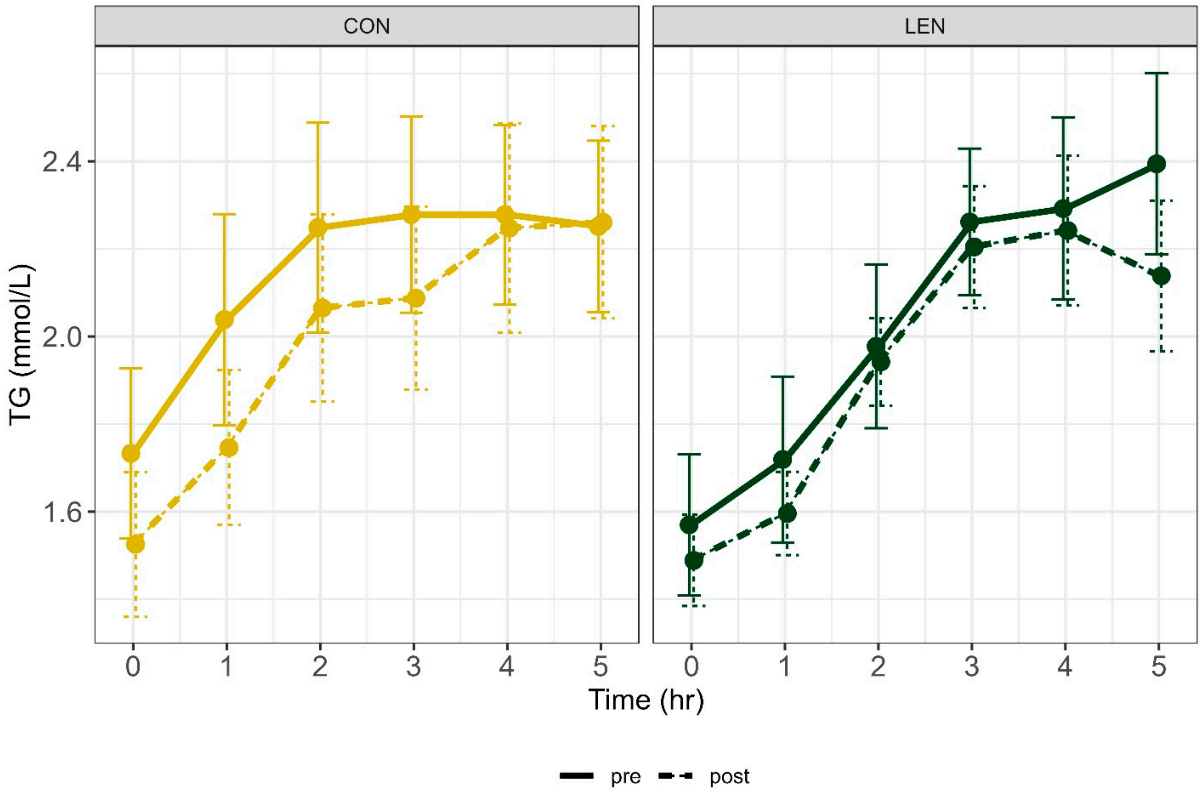

Supplementary Figure S7. Postprandial glucose (GLU) response to a 50 gram, high-fat meal challenge by meal group before and after the 12-week dietary intervention. Points indicate group means, bars represent ± standard error. LEN, lentil; CON, control.

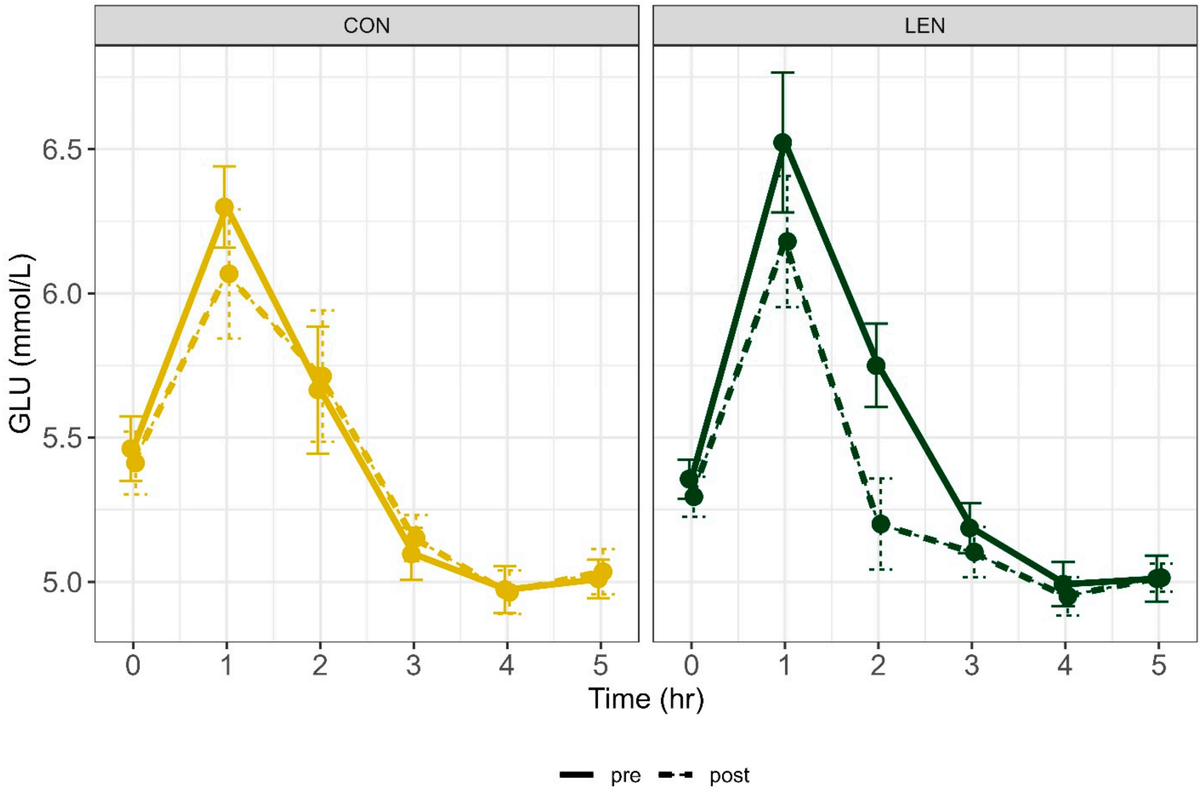

Supplementary Table S1. Nutrient and functional analysis of whole green lentils used in the 12-week intervention.

**Analysis**	**Amount**
Nutrient (per 100g)	
Calories (kcals)	362
Moisture (g)	9.5
PRO (g)	23.4
Fat (g)	2.1
Ash (g)	2.7
Total CHO (g)	62.3
Soluble Fiber (%)	0.4
Insoluble Fiber (%)	13.3
Starch (g)	36.27
Functional	
Foam Expansion (%) (5% w/w)	218
Water Holding Capacity (g/g)	1.3
Foam Stability (%)	94
pH (5% w/w)	6.3

Abbreviations: Kcal, kilocalories; PRO, protein; g, grams; CHO, carbohydrate.

Supplementary Table S2. Overview of intervention meals and their nutrient profile. Profile based off the first week of study meals, where participants received one of each of the seven study meals. Macronutrient totals were rounded to nearest whole number.

	CON					LEN				
	**Kcal**	**PRO g**	**CHO g**	**FAT g**	**Fiber g**	**Kcal**	**PRO g**	**CHO g**	**FAT g**	**Fiber g**
**Bolognese**	260	18	31	16	3	384	23	57	9	10
**Curry**	555	28	63	20	2	413	21	50	9	9
**Loaf**	396	23	38	19	4	482	25	66	9	11
**Taco**	455	25	33	26	3	604	34	76	13	11
**Soup**	272	20	25	5	1	251	18	42	2	7
**Shepherd's Pie**	587	34	53	31	4	549	23	73	21	10
**Chili**	234	14	24	10	2	259	17	36	3	8
**TOTALS**	2,759	162	267	127	19	2,942	161	400	66	66

Abbreviations: CON, control; LEN, lentil.; Kcal, kilocalories; PRO, protein; g, grams; CHO, carbohydrate.

Supplementary Table S3. Change in fasting and postprandial inflammation by meal group before and after the 12-week dietary intervention. Fasting values represent timepoint 0 (fasting) collected prior to 50 gram, high-fat meal challenge. Postprandial values summarized as net area under the curve (AUC) using fasting and 1-, 2-, 3-, 4-, and 5- hour postprandial measurements. Delta values calculated as (post-intervention – pre-intervention) and expressed by Δ Baseline to represent changes in fasting inflammation and Δ AUC to represent changes in postprandial inflammation. Values as mean (standard error). Bold p-values indicate strong evidence of a difference between meal groups.

	**CON (n=18)**		**LEN (n=20)**		**p-value**	
**Cytokine**	**Δ Baseline**	**Δ AUC**	**Δ Baseline**	**Δ AUC**	**Δ Baseline**	**Δ AUC**
IL-6	0.26 ± 1.79	0.09 ± 3.93	2.20 ± 5.52	-12.42 ± 31.92	0.63	0.10
IL-1β	0.04 ± 0.78	1.09 ± 3.92	0.65 ± 2.05	-4.06 ± 9.94	0.19	**0.03**
IL-17	-0.84 ± 4.93	13.82 ± 31.64	3.56 ± 12.62	-27.07 ± 83.07	0.91	**0.04**
IL-23	-12.27 ± 129.20	122.67 ± 731.23	-28.54 ± 527.21	-234.36 ± 941.87	0.74	0.14
IL-10	0.62 ± 12.72	8.69 ± 38.50	3.46 ± 12.93	-43.64 ± 133.11	0.25	0.10
TNF-α	-0.76 ± 1.88	3.07 ± 9.37	1.47 ± 4.17	-7.59 ± 21.85	0.50	0.29
GM-CSF	-4.53 ± 17.42	-77.16 ± 482.39	3.00 ± 24.99	-24.35 ± 67.97	0.11	0.14
IFN-γ	11.67 ± 85.84	6.99 ± 434.06	9.69 ± 143.72	-27.20 ± 438.32	0.44	0.88

Data represent mean ± standard error. Linear mixed modeling used to determine differences ($p < 0.05$) between group means.
Abbreviations: CON, control; LEN, lentil.; IL, interleukin; TNF-α, tumor necrosis factor alpha; GM-CSF, granulocyte macrophage colony stimulating factor, IFN-γ, interferon-gamma; AUC, area under the curve.
